# A new generation computerised metacognitive cognitive remediation programme for schizophrenia (CIRCuiTS): a randomised controlled trial

**DOI:** 10.1017/S0033291717001234

**Published:** 2017-09-04

**Authors:** C. Reeder, V. Huddy, M. Cella, R. Taylor, K. Greenwood, S. Landau, T. Wykes

**Affiliations:** 1 Institute of Psychiatry, Psychology and Neuroscience (IoPPN), King's College London, UK; 2 University College, London, UK; 3 Sussex Partnership NHS Foundation Trust, Sussex, UK; 4 School of Psychology, University of Sussex, Falmer, UK

**Keywords:** Schizophrenia, psychosis, cognitive remediation, neuropsychology, metacognition, cognition, social functioning

## Abstract

**Background:**

Cognitive remediation (CR) is a psychological therapy, which improves cognitive and social functioning in people with schizophrenia. It is now being implemented within routine clinical services and mechanisms of change are being explored. We designed a new generation computerised CR programme, CIRCuiTS (Computerised Interactive Remediation of Cognition – a Training for Schizophrenia), to enhance strategic and metacognitive processing, with an integrated focus on the transfer of cognitive skills to daily living. This large trial tested its feasibility to be delivered in therapist-led and independent sessions, and its efficacy for improved cognitive and social functioning.

**Methods:**

A two arm single blind randomised superiority trial comparing CIRCuiTS plus treatment-as-usual (TAU) with TAU alone in 93 people with a diagnosis of schizophrenia. Cognitive, social functioning and symptom outcomes were assessed at pre- and post-therapy and 3 months later.

**Results:**

85% adhered to CIRCuiTS, completing a median of 28 sessions. There were significant improvements in visual memory at post-treatment (*p* = 0.009) and follow-up (*p* = 0.001), and a trend for improvements in executive function at post-treatment (*p* = 0.056) in favour of the CIRCuiTS group. Community function was also differentially and significantly improved in the CIRCuiTS group at post-treatment (*p* = 0.003) but not follow-up, and was specifically predicted by improved executive functions.

**Conclusions:**

CIRCuiTS was beneficial for improving memory and social functioning. Improved executive functioning emerges as a consistent predictor of functional gains and should be considered an important CR target to achieve functional change. A larger-scale effectiveness trial of CIRCuiTS is now indicated.

## Introduction

Cognitive dysfunction is a hallmark of a diagnosis of schizophrenia, a good predictor of functional recovery (Green *et al.* 2000) and consequently a valued treatment target (Wykes & Spaulding, 2011). Cognitive remediation (CR) is ‘a behavioural training-based intervention to improve cognitive processes (e.g. attention, memory, executive functioning), with the general aim of durable benefits on community functioning’ (CREW, 2012). Meta-analytic results demonstrate beneficial effects on cognition and functioning (Krabbendam & Aleman, 2003; McGurk *et al.* 2007; Wykes *et al.* 2011), although generalisation to functional benefits are frequently restricted to strategy-based, rather than drill-and-practice, CR approaches, delivered in the context of vocational rehabilitation (Wykes *et al.* 2011; Drake *et al.* 2014). There is consensus, with some supporting evidence that cognitive improvements are likely to be maximised if the CR includes (i) massed practice (i.e. highly repetitive practice taking place on several days a week for prolonged periods), (ii) scaffolded learning facilitating high success rates, and (iii) a focus on motivation (Wykes & Reeder, 2005; Wykes & Spaulding, 2011; Vinogradov *et al.* 2012). CR programmes have generally not been purpose-built and frequently do not use evidence-based principles to drive cognitive change, or to generalise cognitive changes to functioning. The lack of an optimal, easy-to-deliver CR programme is notable, given that CR is increasingly being adopted in governmental guidelines (SIGN, 2013) and routine clinical practice (New York State Office of Mental Health, 2010).

Our group has developed a new generation, computerised metacognitive CR programme, CIRCuiTS [Computerised Interactive Remediation of Cognition – a Training for Schizophrenia (Reeder & Wykes, 2010)], fit for widespread clinical dissemination, which uses evidence-based cognitive training principles, and targets functioning directly. Its focus on developing metacognition [i.e. thinking about thinking (Flavell, 1979)] is underpinned by a model that suggests that the transfer of cognitive skills to daily activities depends on metacognitive knowledge and metacognitive regulation, or the ability to effectively understand and manage one's own cognitive processes (Wykes & Reeder, 2005). This entails a strategy-based approach, which is supported by studies showing that changes in executive function (i.e. metacognitive regulation) better predict functional change in schizophenia than changes in other cognitive processes such as memory (Reeder *et al.* 2006, 2014; Eack *et al.* 2009; Wykes *et al.* 2012).

CIRCuiTS was designed for people with a schizophrenia diagnosis and developed with service user and therapist involvement. It is delivered by a therapist, supplemented by independent sessions. It is highly acceptable to service users and clinicians (Reeder *et al.* 2015). An independent randomised controlled trial comparing CIRCuiTS plus Cognitive Behavioural Therapy for psychosis (CBTp) with social contact plus CBTp (Drake *et al.* 2014) showed that CIRCuiTS participants achieved the same symptom improvements with significantly fewer CBTp sessions and signficantly greater insight and executive improvements.

The current randomised controlled trial (RCT) compares CIRCuiTS plus treatment-as-usual (TAU) with TAU alone in people with schizophrenia. Our objectives were to assess (i) the feasibility of delivering CIRCuiTS with therapist-led sessions supplemented by independent working; and (ii) the efficacy of CIRCuiTS for improved cognition and social functioning.

## Method

Ethical permission reference number 08/H0807/26.

### Design

A two arm randomised superiority trial comparing CIRCuiTS plus TAU with TAU alone. Outcomes were measured at week 0 pre-randomisation (baseline), week 12 (post-treatment) and week 26 (follow-up).

### Participants

Inclusion criteria were (i) DSM-IV diagnosis of schizophrenia or schizo-affective disorder, (ii) at least 1 year's contact with mental health services, (iii) 17–65 years, and (iv) performance more than one s.d. below the normative mean in working memory [digit span (Wechsler, 1993)] and/or cognitive flexibility [Wisconsin Card Sorting Test (WCST) (Heaton *et al.* 1993) or Hayling Sentence Completion Test (Burgess & Shallice, 1997)]. The protocol criterion of poor social function was interpreted as not being in paid employment, receiving financial benefits for disability, or not living independently, due to difficulties in finding an informant for the pre-specified questionnaire. This criterion was included since social functioning is a target of the intervention and a secondary outcome. Therefore, participants needed to show room for improvement in this respect. Exclusion criteria were (i) plans to change medication during the study, (ii) substance dependence or (iii) evidence of an organic cause to cognitive difficulties.

Participants were recruited across the UK South London and Maudsley Mental Health National Health Service (NHS) Foundation Trust. Following an explanation of the study, written informed consent was obtained from all participants.

### Interventions

#### Treatment-as-usual

Routine psychiatric care provided within the UK National Health Service, which may have taken place within community, inpatient or rehabilitation settings. In all settings, this is likely to include individualised multi-disciplinary contacts such as medication review and monitoring by a psychiatrist, regular meetings with a mental health nurse for support, and less frequently, psychological or occupational therapy, residential support with self-care, and attendance at day centres or rehabilitation programmes.

### CR programme [CIRCuiTS (Reeder & Wykes, 2010; Reeder *et al.* 2015)]

CIRCuiTS is a web-based computerised CR therapy, delivered by a therapist but supplemented with independent sessions to facilitate massed practice. It targets metacognition, particularly strategy use, in addition to providing massed practice of basic cognitive functions. The therapist facilitates motivation, metacognitive and strategy development and generalisation of learning by encouraging the participant to learn about and regulate their cognitive performance and to transfer this learning to meet real-world goals. Therapists provide additional scaffolding for CR tasks to ensure consistent successful performance. Independent sessions involve carrying out cognitive tasks allocated by the therapist to ensure scaffolded learning.

Real-world cognitive goals are set collaboratively, and then CIRCuiTS tasks are used to identify cognitive strengths and difficulties and factors affecting cognitive performance. The primary cognitive targets are attention, memory and executive functioning and repetitive tasks gradually increase in difficulty in line with individual highly successful performance. Participants develop a set of personalised strategies to improve their cognitive performance, and achieve their goals.

The CR tasks are either ‘abstract’ (neutral content, such as numbers, and designed to target specific cognitive functions) or ‘exercises’ (cognitively complex and ecologically valid) associated with work, social situations, cooking, shopping and travelling. (Please see the online Supplementary material 1 for some examples). Therapists encourage participants to apply the skills learnt to daily life and to practice *in vivo,* in order to achieve their real-world goals. Thus, functional outcomes are directly targeted by the therapy.

#### Rate of delivery

CIRCuiTS was offered at least three times a week (maximum 12 weeks), up to 40 sessions lasting up to an hour. Where possible, according to participants’ ability and choice, therapists encouraged them to carry out additional independent sessions (please see online Supplementary material 1 for further information).

### Therapists and therapy fidelity

Therapists were supervised, trained graduate psychologists. A high degree of fidelity is ensured using computerised delivery but audio-recordings of three sessions (from start, middle and end of therapy) for all participants who consented to recordings (*n* = 28 sessions) were also rated using a modified CRT Fidelity Scale (Stenmark, 2006) (see online Supplementary materials 2).

### Outcome measures

Participants were reimbursed £5 per hour for assessments.

#### Baseline assessments

*Socio-demographic and clinical variables* collated from participants, case notes and mental health workers.

*Estimated premorbid full scale IQ:* Wechsler Test of Adult Reading (Wechsler, 2001).

*Estimated current IQ* [*pro-rated* (Silverstein, 1982)]: Vocabulary and Block Design from the Wechsler Adult Intelligence Scale – Third Edition – UK [WAIS-III-UK (Wechsler, 1993)].

*Symptoms:* Positive and Negative Syndrome Scale (Kay *et al.* 1987) (PANSS) (total score). A 30-item clinical interview to assess symptom severity for schizophrenia, administered by trained graduate psychologists achieving high inter-rater reliability to an expert trainer. Positive, negative, disorganised, excited and depressed subscales were used (Wallwork *et al.* 2012).

#### Primary outcomes

The primary point of interest was 12 weeks (post-therapy).

*Verbal working memory:* Digit Span [WAIS-III-UK (Wechsler, 1993)], a working memory task: total raw score (high scores – good performance).

*Visual memory:* Rey Osterreith Complex Figure (ROCF) (Rey, 1941; Osterreith, 1944), a visual memory test: immediate recall raw score (high scores – good performance).

*Verbal executive function:* Hayling Sentence Completion test (Burgess & Shallice, 1997), measuring response inhibition: total scaled score (high scores – good performance).

*Visual executive function:* WCST (Heaton *et al.* 1993), testing abstraction and cognitive flexibility: percentage errors raw score (high scores – poor performance).

#### Secondary outcomes

*Community functioning:* Time Use Survey [UK 2000 Time Use Survey (Short, 2006)]. A semi-structured interview recording participants’ time use, selected to capture widely disparate clinically meaningful increases in functional activity. Key outcome: total hours per week over the past month spent in employment, education, voluntary work, voluntary and structured leisure activities, housework and chores, childcare, sports and hobbies.

*Symptoms:* PANSS: positive, negative and disorganised symptom subscales (high scores – high symptom levels).

### Sample size

Following the most recent meta-analysis (Wykes *et al.* 2011), the planned sample size was revised to 44 per group allowing detection of an effect size of 0.6 or larger at post-treatment with 80% power using an independent samples *t* test at the 5% significance level. Assuming a drop out rate of 10%, 49 participants per group were needed.

### Randomisation and blinding

Following the initial assessment, consecutive referrals of participants meeting inclusion criteria were allocated (1:1) to CIRCuiTS plus TAU or TAU using an online system, independently set up by the Clinical Trials Unit, KCL. A minimisation algorithm was used to ensure balance in terms of the gender and age group (above and below 40 years) stratifiers.

Graduate psychologists blind to group assignment conducted all assessments. All the analyses not requiring group identification were carried out blind to allocation.

### Statistical analyses

#### Therapy feasibility analyses (conducted by CR)

These summarised therapy adherence (number and length of completed sessions, including independent sessions) for all CIRCuiTS participants. We judged 20 sessions *a priori* to constitute a minimum therapy course. Therapy completers and non-completers and those who did and did not complete independent sessions were compared on age, current IQ, five symptom dimensions and the primary cognitive outcomes at baseline using *t* tests or Mann–Whitney *U* tests.

#### Primary and secondary outcome group comparisons

Formal analyses were carried out on an intention-to-treat basis by SL to evaluate the efficacy of CIRCuiTS in terms of primary and secondary outcomes at 12 and 26 weeks.

Linear mixed models fitted by maximum likelihood (ML) simultaneously modelled the 12 and 26 week data. The models were parameterised to provide separate group effect estimates at 12 and 26 weeks (see Table 3) and effect estimates standardised by dividing by respective baseline s.d.s. Models include (fixed) effects of time, trial arm and a group × time interaction. Models always include randomisation stratifiers and baseline values of the variable under investigation as a covariate to increase power. They further conditioned on variables that were found to predict attrition to make more realistic assumptions regarding the missing data mechanism. (The resulting ML estimates are valid under the missing at random assumption). To detect such variables empirically a logistic regression was conducted with the dependent variable ‘missingness of the primary outcome variables at 26 weeks’. This used a forward selection approach (inclusion threshold 10%) to test whether any of: PANSS five factor scores, ethnic group, employment status, estimated premorbid and current IQ, or baseline chlorpromazine equivalent, predicted missingness, in addition to age and gender. PANSS excited scores and premorbid IQ were found to be predictive and hence included as covariates in all analysis models. Finally linear mixed models contained a randomly varying intercept at the level of the participant to account for correlation between the two repeated measures.

#### Exploratory mechanism analyses

Therapy characteristics were correlated with change in each of the four primary cognitive outcomes and community functioning over 12 weeks for CRT completers: (i) total number of sessions completed, (ii) mean number of tasks completed per session, (iii) mean number of strategies, rated with high usefulness, used per session, and (iv) whether or not independent sessions were completed.

To explore whether change in any of the cognitive variables singly partially mediated the effect of CIRCuiTS on the functioning outcome we followed a Baron–Kenny approach (Baron & Kenny, 1986; MacKinnon & Valente, 2014). We adjusted mediator and outcome models for covariates identified in the efficacy analyses.

## Results

**Fig. 1.**
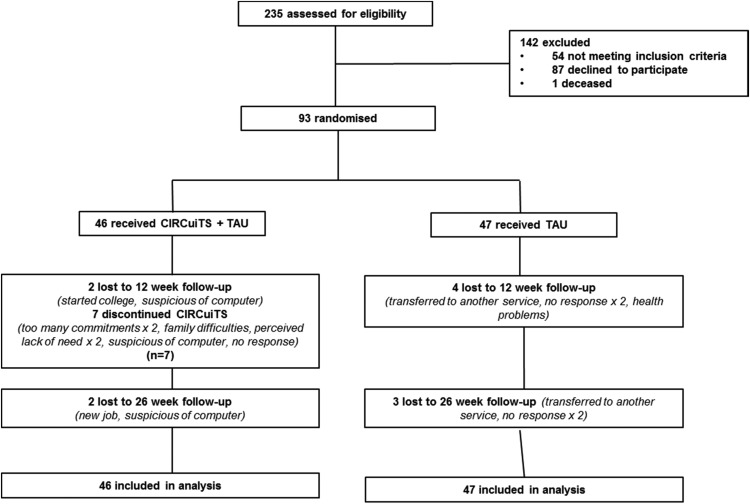
Consort diagram.

In total 93 people were randomised between 24th May 2010 and 29th May 2012. The final follow-up assessment was on 26th November 2012 (see Fig. 1).

### Participant characteristics

Randomisation was successful in balancing the trial arms with regard to baseline variables (see Table 1).

**Table 1.** Participant characteristics

Characteristic	Complete sample (*n* = 93) mean (s.d.)/frequency (%)	Group receiving CIRCuiTS (*n* = 46)	Group not receiving CIRCuiTS (*n* = 47)
Age	38.3 years (10.4 years)	38.7 years (10.1 years)	37.9 years (10.9 years)
Gender			
Women	33 (35.5%)	14 (30.4%)	19 (40.4%)
Men	60 (64.5%)	32 (69.6%)	28 (59.6%)
Years in education	13.2 years (2.5 years)	13.5 years (2.6 years)	13.0 years (2.4 years)
Marital status			
Single	77 (82.8%)	39 (84.8%)	38 (80.9%)
Married	7 (7.5%)	3 (6.5%)	4 (8.5%)
Separated/divorced	9 (9.7%)	4 (8.7%)	5 (10.6%)
Estimated premorbid IQ	93.5 (10.8)	94.2 (10.5)	92.8 (11.2)
Current employment			
Paid or self employment	6 (6.5%)	3 (6.5%)	3 (6.4%)
Voluntary employment	16 (17.2%)	6 (13.0%)	10 (21.3%)
Unemployed	58 (62.3%)	29 (63.0%)	29 (61.7%)
Student	10 (10.8%)	6 (13.0%)	4 (8.5%)
Domestic responsibilities	2 (2.1%)	1 (2.2%)	1 (2.1%)
Other	1 (1.1%)	1 (2.2%)	0 (0.0%)
Current accommodation			
Independent accommodation	52 (55.9%)	23 (50.1%)	29 (61.7%)
Staffed accommodation	24 (25.8%)	14 (30.4%)	10 (21.2%)
Unstaffed group accommodation	3 (3.2%)	1 (2.2%)	2 (4.3%)
Acute psychiatric ward	1 (1.1%)	0 (0.0%)	1 (2.1%)
Rehabiliation psychiatric ward	13 (14.0%)	8 (17.4%)	5 (10.6%)
Time since first psychiatric contact			
Less than 1 year	4 (4.3%)	1 (2.2%)	3 (6.4%)
1–5 years	16 (17.2%)	8 (17.4%)	8 (17.0%)
5–10 years	19 (20.4%)	4 (8.7%)	15 (31.9%)
More than 10 years	54 (58.1%)	33 (71.7%)	21 (44.7%)
Ethnicity			
White	23 (24.7%)	13 (28.3%)	10 (21.3%)
Black	54 (58.1%)	25 (54.3%)	29 (61.7%)
Asian	6 (6.5%)	2 (4.3%)	4 (8.5%)
Mixed race	10 (10.8%)	6 (13.0%)	4 (8.5%)
PANSS			
Positive	8.5 (4.5)	8.3 (4.2)	8.7 (4.8)
Negative	10.8 (4.9)	11.2 (5.2)	10.5 (4.6)
Disorganised	8.0 (3.0)	8.1 (3.3)	8.0 (2.6)
Excited	5.3 (1.8)	5.4 (2.1)	5.1 (1.6)
Depressed	6.9 (3.2)	6.9 (3.3)	6.8 (3.1)
Medication			
Typical anti-psychotics	9 (9.7%)	4 (8.7%)	5 (10.6%)
Atypical anti-psychotics	82 (88.2%)	42 (91.3%)	43 (91.5%)
Risperidone	17 (18.3%)	9 (19.6%)	8 (17.0%)
Olanzapine	18 (19.4%)	9 (19.6%)	9 (19.1%)
Clozapine	30 (32.3%)	14 (30.4%)	16 (34.0%)
Chlorpromazine equivalent dosage	Median 333.3 mg (0–1920.0 mg)	Median 326.6 mg (0–1920.0 mg)	Median 377.5 mg (43.8–1800.0 mg)

Only four participants were completely lost to follow-up (i.e. 4.3% at both 12 and 26 week assessment time points, Fig. 1). 17 participants had a missing value for at least one of the four primary outcome variables at 26 weeks (18.3%).

### CIRCuiTS feasibility

Of the seven (15%) non-completers, six completed only one or two sessions, and one completed 16 sessions. For all CIRCuiTS participants, the median number of sessions completed was 25.5 (range 1–41). Amongst completers, the median was 27.5 (20–41), the mean session length was 45.5 min (s.d. 10.2), a mean of 4.8 (s.d. 1.6) tasks per session were completed and a mean of 7.1 (s.d. 4.2) useful strategies used per session.

Nine people (20%) completed at least one independent session (median 6, 1–10). Participants who completed independent sessions completed a similar number of sessions overall (median 27.5, 20–41) to those who did not complete independent sessions (median 27.0, 20–40). The only significant difference (*t* = 2.8, df = 39.6, *p* = 0.007), with little clinical importance, between those completing independent sessions and those who did not, was on the PANSS excited score: independent sessions mean = 5.68, s.d. = 2.2; no independent sessions mean = 4.44, s.d. = 0.73).

Five therapists conducted the CRT with three seeing fewer than 10 patients. The majority of rated sessions (18 sessions – 64.2%) were scored 7/7 on the modified CRT Fidelity Scale and the lowest score (only three sessions – 10.7%) was 5/7.

### Does CIRCuiTS lead to improved cognitive and functioning outcomes?

Table S1 (please see online Supplementary material 3) summarises observed primary and secondary outcomes.

Table 2 shows the results of the formal statistical analyses. Since we had four primary outcomes, the significance level was adjusted (*α* = 0.05/4 = 0.0125). We found significant improvements for immediate visual memory at post-treatment (*p* = 0.009) and at follow-up (*p* = 0.001), and a trend for improvement in non-verbal executive functioning at post-treatment (*p* = 0.056), in favour of CIRCuiTS. The secondary outcome analyses demonstrated that CIRCuiTS participants spent significantly more time in structured activities at post-treatment (*p* = 0.003). There was also some evidence (*p* = 0.049) that PANSS positive symptoms were lower in the CIRCuiTS arm at post-treatment.

**Table 2.** Estimated treatment group effects at 12 and 26 weeks post randomisation

	12 weeks		26 weeks	
Outcome	*z*-statistic (*p* value)	Estimated difference (TAU-CR) [95% CI] Stand. effect size	*z*-statistic (*p* value)	Estimated difference (TAU-CR) [95% CI] Stand. effect size
*Primary outcomes*				
Verbal working memory (Digit span)	−1.19 (*p* = 0.24)	−0.564 [−1.494 to 0.366] ES = −0.16	−0.99 (*p* = 0.32)	−0.474 [−1.417 to 0.464] ES = −0.13
Visual memory (ROCF)	−2.63 (*p* = 0.009)	−2.403 [−4.194 to −0.611] ES = −0.35	−3.46 (*p* = 0.001)	−3.166 [−4.957 to −1.374] ES = −0.46
Verbal executive function (Hayling)	−0.65 (*p* = 0.52)	−0.421 [−1.699 to 0.857] ES = −0.09	−0.83 (*p* = 0.41)	−0.545 [−1.839 to 0.749] ES = −0.12
Visual executive function (WCST)	1.91 (*p* = 0.056)	6.531 [−0.176 to 13.237] ES = 0.36	1.66 (*p* = 0.098)	5.713 [−1.046 to 12.473] ES = 0.32
*Secondary outcomes*				
Time spend in structured activities^a^	−3.01 (*p* = 0.003)	0.622^a^ [0.457–0.847]^a^ ES = −0.55	−0.42 (*p* = 0.67)	0.936^a^ [0.687–1.276]^a^ ES = −0.08
Positive symptoms^a^ (PANSS)	1.97 (*p* = 0.049)	1.129^a^ [1.001–1.275]^a^ ES = 0.24	0.26 (*p* = 0.80)	1.016^a^ [0.899–1.149]^a^ ES = 0.03
Negative symptoms^a^ (PANSS)	−0.21 (*p* = 0.83)	0.986^a^ [0.870–1.119]^a^ ES = −0.03	0.47 (*p* = 0.63)	1.031^a^ [0.908–1.172]^a^ ES = 0.08
Disorganised symptoms^a^ (PANSS)	1.28 (*p* = 0.20)	1.073^a^ [0.964–1.194]^a^ ES = 0.20	1.81 (*p* = 0.07)	1.106^a^ [0.992–1.233]^a^ ES = 0.28

a Outcome was analysed on the ln-scale due to positive skewness. Unstandardised effect estimates represent multiplicative (factor) effects and need to be compared with the factor value ‘1’ (=no group effect).

### Are aspects of therapy associated with cognitive and functional outcomes?

More completed sessions were associated with greater non-verbal executive improvement (*r* = −0.31) at 12 weeks and a larger benefit for structured activity (*r* = 0.22). Improvement in visual memory was associated with more tasks completed and a higher number of useful strategies (*r* = 0.39 and *r* = 0.24 respectively). Completion of independent sessions was not associated with any outcome.

### Does cognition mediate the CR effect on functioning?

The exploratory mediation analyses are summarised in Table 3. Change in only one of the four primary cognitive outcomes, the WCST, showed a significant association with increased time in structured activities at 12 weeks (estimated standardised regression coefficient −0.28, 95% CI from −0.51 to −0.06). Approximately 20% of the increase in (ln-)structured time in the CIRCuiTS arm was mediated by a reduction in WCST errors.

**Table 3.** Mediation of CR effects on time spend in structured activities at 12 weeks by primary cognitive outcomes (n = 87)

Putative mediator	Estimated total effect of CR on functioning (TAU-CR) [95% CI]^a^	Estimated effect of CR on mediator (TAU-CR) [95% CI]	Estimated effect of mediator on functioning (per baseline s.d.) [95% CI]	Direct (non-mediated) effect of CR on functioning (TAU-CR) [95% CI]^a^	Indirect (mediated) effect of CR on functioning (TAU-CR) [95% CI]^b^ (*p* value)^c^	% mediated
Verbal working memory (Digit span)	−0.58 [−0.96 to −0.21]	−0.18 [−0.44 to 0.09]	−0.11 [−0.43 to 0.20]	−0.60 [−0.98 to −0.23]	0.02 [−0.05 to 0.10] (*p* = 0.53)	n.a.^d^
Visual memory (ROCF)	−0.58 [−0.95 to −0.21]	−0.35 [−0.61 to −0.10]	−0.05 [−0.38 to 0.28]	−0.60 [−0.99 to −0.21]	0.02 [−0.08 to 0.19] (*p* = 0.76)	n.a.^d^
Verbal executive function (Hayling)	−0.57 [−0.94 to −0.17]	−0.08 [−0.39 to 0.23]	0.16 [−0.11 to 0.43]	−0.56 [−0.93 to −0.18]	−0.01 [−0.16 to 0.02] (*p* = 0.64)	2.3%
Visual executive function (WCST)	−0.53 [−0.91 to −0.15]	0.37 [−0.01 to 0.75]	−0.28 [−0.51 to −0.06]	−0.43 [−0.80 to −0.05]	−0.11 [−0.31 to −0.01] (*p* = 0.12)	19.8%

a Time spend in structured activities was ln-transformed and then standardised to express functioning in units of baseline standard deviations (s.d.s).

b Confidence interval for direct effect is bias corrected bootstrap interval (using 1000 bootstrap replicates) as recommended in (MacKinnon & Valente, 2014).

c Sobel test for zero mediation.

d Not applicable when the direct and indirect CR effect point in different directions.

## Discussion

### CIRCuITS feasibility

This study demonstrates that CIRCuiTS, a new generation computerised metacognitive CR programme, is feasible for people with a schizophrenia diagnosis with cognitive impairment. 85% of participants offered CIRCuiTS attended at least 20 sessions within 12 weeks. This adherence rate compares favourably with other CR studies (Wykes *et al.* 2011), including computerised CR (Murthy *et al.* 2012). Six of the seven participants with poor adherence stopped attending after only one or two sessions, suggesting that for most participants engagement was achieved very quickly.

The target dose was 40 sessions but the median number for completers was 28. The average attendance of approximately two sessions per week is consistent with attendance rates in our previous trials, which have generally used extended time periods to achieve a higher dose (Wykes *et al.* 2007). One meta-analysis of CR (McGurk *et al.* 2007) reported a mean intensity of 2 hours per week, and this remains a common regime in CR trials (Bowie *et al.* 2012; Drake *et al.* 2014). The persistence of a two session per week norm, despite the emphasis on massed practice in CR programmes, may reflect the clinical reality that motivating people with schizophrenia [known to often have motivational impairments (Cella *et al.* 2014)], to attend more than twice a week is challenging.

### Does CIRCuiTS lead to improved cognitive and functional outcomes?

Both post-therapy and at 3 month follow-up the CIRCuiTS group showed significantly greater improvement in immediate visual memory. This is encouraging in light of findings of deterioration in visual-spatial/constructional skills over 3 years in a sample of people with chronic schizophrenia (Dickerson *et al.* 2014): CIRCuiTS may protect against cognitive decline. There was also a trend (*p* = 0.056) for greater improvement in WCST scores following CIRCuiTS, which may be important since this was the main cognitive driver of functioning improvement.

Changes in other cognitive outcomes were not significantly different between groups. We have noted that the mean number of sessions was lower than intended and consequently may have been insufficient for consistent cognitive improvements. In fact, greater WCST and social functioning improvements at post-treatment were associated with doing more therapy sessions. The main theoretical change mechanism for CIRCuiTS, in addition to massed practice, is via the development of metacognitive knowledge and metacognitive regulation, including the use of strategies for a more systematic and organised approach to tasks. Greater improvement in immediate visual memory was predicted by a higher mean number of tasks carried out within sessions and a higher mean number of strategies rated as helpful by patients. This is consistent with massed practice and strategy use being the chief mechanisms of cognitive change. However, note that our study only estimates associations with aspects of therapy, which are not necessarily causal.

A more strategic approach is likely to entail a considerable shift in the way in which tasks are undertaken, and this may lead to an initial deterioration in performance (Harvey *et al.* 2009). The two cognitive tasks, which did not show improvement require immediate, rapid responses, and so would not have been likely to benefit from an increase in strategic thinking, which may take more time. However, better strategy use does appear to underpin more efficient executive and memory performance in schizophrenia in both the WCST (Choi & Kurtz, 2009) and the ROCF (Landgraf *et al.* 2011), consistent with the cognitive improvements in this study.

To assess functional changes, we used a Time Use Survey measure in an attempt to capture the wide range of changes (from gaining paid employment to beginning to meet with a relative once or twice a week) that may be meaningful within a sample of people with a schizophrenia diagnosis. CIRCuiTS led to improved community functioning post-therapy by increasing the hours spent in structured activity, although this was not sustained at follow-up. This presumably reflects the constraints of offering therapy within a research context. For many people, sustained improvement and recovery requires maintained support. This is consistent with findings that CR is most beneficial when offered in the context of an adjunctive rehabilitation programme (Wykes *et al.* 2011).

### Does cognition mediate improvements in functioning?

Only improved executive functioning was associated with benefits for functioning: this finding is well-supported in the literature (Reeder *et al.* 2006, 2014; Eack *et al.* 2009; Wykes *et al.* 2012) and is consistent with the metacognitive model, which underpins CIRCuiTS (Wykes & Reeder, 2005). Executive functions are likely to be important CR targets to achieve functional change. However, note that we cannot establish causality at this stage. Our mediation models were exploratory in nature and make a number of assumptions; including that there are no further hidden confounders of the cognition-functioning relationship and that measurement error in cognitive variables is negligible.

### Study limitations

Despite being one of the largest CR trials to date, our final sample size might have been too low to identify moderate effects at the 5% significance level taking into account our multiple outcome comparisons. Consequently, we may have failed to detect some effects of CIRCuiTS.

We did not include an active control condition: a lack of agreement regarding what constitutes specific *v*. non-specific effects of CR, combined with evidence that active computerised CR controls may not be effective (Gomar *et al.* 2015), made it difficult to justify public funding support for an additional control treatment arm.

### Conclusions

CIRCuiTS, a new generation computerised CR programme, is feasible to deliver both with therapist-led and independent sessions. It led to improved performance in immediate visual memory which relies on executive organisational skills for effective encoding, and this improvement was maintained at 3-month follow-up. It also resulted in increased structured activity post-therapy. A large-scale effectiveness trial is warranted.

## Supplementary material

For supplementary material accompanying this paper visit http://dx.doi.org/10.1017/S0033291717001234. click here to view supplementary material
