# Supplementary material for: A new generation computerised metacognitive cognitive remediation programme for schizophrenia (CIRCuiTS): a randomised controlled trial
Source: Psychol Med. 2017 Sep 4;47(15):2720–30. doi: 10.1017/S0033291717001234 (PMC5647677; doi:10.1017/S0033291717001234)
Supplement: Supplementary file 1 [file S0033291717001234sup001.doc]

**Supplementary material 1**

**Example of an abstract task – ’Image Copy’**

In this task requiring visual attention switching, the participant drags the icons from the ’tools’ in the play area to make a copy of the image. The therapist encourages the participant to choose from a list of strategies (suggested by the computer at first, and later self-generated), and then to use, monitor and evaluate this strategy as they complete the task.

**Screenshot of ’Image Copy’ abstract task**


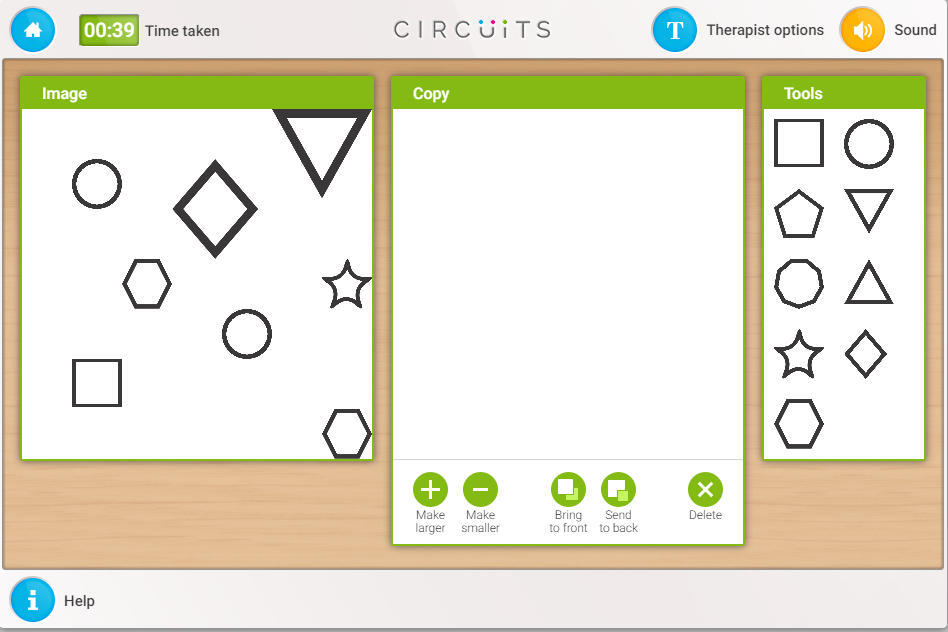


**Example of an exercise – ’Track your position’**

In this more complex task, requiring visual attention switching as above, but also visual working memory to change perspectives, the participant is asked to follow their journey through the village, which is shown in moving animation in the left hand image, and when the image stops, to select their location on the map on the right. Again, strategy use is an integral part of the task.

**Screenshot of ’Track your position’ exercise**


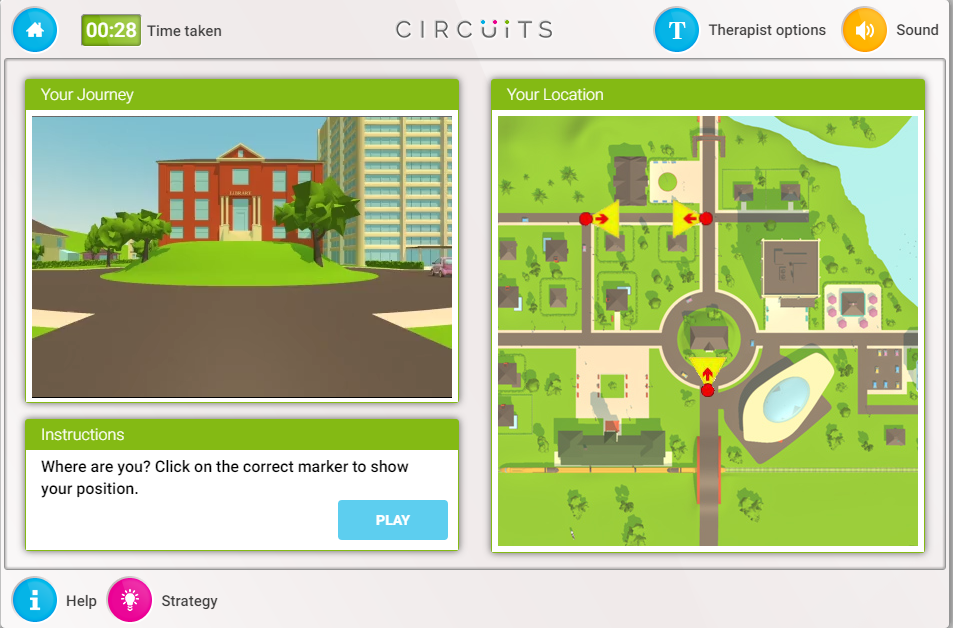


**Delivering independent sessions**

Independent sessions were conducted by the participant away from the therapist, usually at the participants’ home or in a public place (e.g. a library) offering computer access. The therapist and participant would discuss an agreed assignment, which would either involve the participant continuing to work through the online CIRCuiTS programme alone, or the therapist would set specific CIRCuiTS homework (consisting of a few of the CIRCuiTS tasks), which could be completed either online or offline, using tasks stored on a memory stick. The therapist was able to view the participants’ work at a later date (either online or by the return of the updated memory stick) and the two would discuss learning points, e.g. noting things that went well or which were difficult.

**Supplementary material 2**

**Fidelity scale modified for CIRCuiTS (based on Stenmark, 2006)**

Rater: Therapist: Session:

Yes No

| *Following the structure of the session (Aim for the session 1/1)* | **Tailoring:** T tailors the tasks and session to match the level and functioning of the patient. Example: ‘you seem to find this task hard, let’s move on for now’. |  |  |
| --- | --- | --- | --- |
| *Positive reinforcement((Aim for the session 1/1)* | **Positive reinforcement:** T reinforces the patient’s effort even if it is difficult for the patient, in an adequate, recurrent and positive way. |  |  |
| Strategies, reduction of information  (*Aim for the session* 2/9) | **Modelling:** T models the preferred strategy ** according to principles, and helps the patient to generalise the strategies. |  |  |
|  | **Verbalisation**: T uses verbalisation of cues, prompts and strategies, according to principles, relating to the task before hand ˆ and helps the patient to generalise it. Se ovan om generalise |  |  |
|  | **Reduction of information:**T shows and encourages the patient to use strategies for reduction of the information in the tasks into smaller pieces |  |  |
|  | **Smaller steps:**T shows and encourages the patient to use strategies for breaking the task at hand into smaller steps |  |  |
|  | **Simplifying:** T shows and encourages the patient to use strategies for simplifying the task at hand |  |  |
|  | **Chunking:** T shows and encourages the patient to use strategies for “chunking” information to be remembered. |  |  |
|  | **Categorising:** T shows and encourages the patient to use strategies for categorisation (categorising to-be-remembered information) |  |  |
|  | **Prompts:** T gives and encourages the patient to use written prompts |  |  |
|  | **Organisation:** T shows and encourages the patient to use strategies for organisation |  |  |
| *Empathy (Aim for the session 5/10)* |  |  |  |
| *Computer* | Session must last at least 20 minutes  At least 4 tasks should have been completed.  At least four strategies should have been used. |  |  |

Table S1. Primary and secondary outcomes by assessment time point and trial arm.

|  | **Baseline** | | | | **Post-therapy** | | | | **Follow-up** | | | |
| --- | --- | --- | --- | --- | --- | --- | --- | --- | --- | --- | --- | --- |
|  | **CIRCuiTS** | | **Controls** | | **CIRCuiTS** | | **Controls** | | **CIRCuiTS** | | **Controls** | |
|  | **n** | **Mean (SD)**  **Median**  **(Range)** | **N** | **Mean (SD)**  **Median**  **(Range)** | **N** | **Mean (SD)**  **Median**  **(Range)** | **n** | **Mean (SD)**  **Median**  **(Range)** | **n** | **Mean (SD)**  **Median**  **(Range)** | **n** | **Mean (SD)**  **Median**  **(Range)** |
| ***Primary outcomes*** | | | | | | | | | | | | |
| Verbal working memory (Digit span) | 46 | 13.8 (3.6)  14.0  (8.0-23.0) | 47 | 13.3 (3.5)  13.0  (6.0-21.0) | 44 | 14.3 (3.8)  14.0  (8.0-22.0) | 43 | 13.4 (3.6)  13.0  (4.0-23.0) | 41 | 14.5 (3.4)  14.0  (7.0-23.0) | 43 | 13.6 (3.9)  13.0  (7.0-23.0) |
| Visual memory (ROCF) | 46 | 10.8 (6.5)  10.7  (.0-28.5) | 47 | 12.1 (7.2)  13.0  (.0-32.0) | 44 | 15.3 (7.7)  15.0  (1.5-31.0) | 43 | 14.0 (7.1)  14.0  (.5-33.0) | 43 | 16.5 (8.5)  15.5  (2.0-34.0) | 44 | 14.6 (7.8)  15.0  (2.5-34.0) |
| Verbal executive function (Hayling) | 46 | 13.4 (5.0)  14.0  (3.0-20.0) | 45 | 13.7 (4.0)  13.0  (3.0-20.0) | 44 | 15.6 (4.5)  17.0  (5.0-22.0) | 41 | 15.4 (4.2)  17.0  (3.0-21.0) | 40 | 15.9 (4.7)  17.0  (3.0-23.0) | 41 | 15.7 (4.2)  17.0  (3.0-21.0) |
| Visual executive function (WCST) | 45 | 51.2 (18.2)  52.0  (18.0-76.0) | 43 | 52.2 (18.0)  53.0  (14.0-79.0) | 43 | 43.3 (21.10)  42.0  (11.0-80.0) | 43 | 51.2 (20.1)  54.0  (14.0-81.0) | 41 | 44.6 (20.7)  47.0  (12.0-75.0) | 41 | 51.2 (20.8)  55.0  (11.0-80.0) |
| ***Secondary outcomes*** | | | | | | | | | | | | |
| Time in structured activity | 46 | 32.1 (19.5)  31.0  (2.3-80.8) | 47 | 38.5 (25.6)  31.5  (4.0-109.6) | 44 | 36.4 (22.7)  34.9  (3.5-102.5) | 43 | 28.4 (21.8)  24.2  (1.0-98.3) | 43 | 32.8 (19.4)  31.0  (1.5-78.8) | 44 | 36.5 (27.8)  28.5  (2.0-136.7) |
| Positive symptoms (PANSS) | 46 | 8.3 (4.2)  8.0  (4.0-18.0) | 47 | 8.7  (4.8)  8.0  (4.0-23.0) | 46 | 7.6 (4.0)  6.0  (4.0-17.0) | 44 | 8.4 (3.9)  8.0  (4.0-20.0) | 41 | 8.0 (4.0)  6.0  (4.0-18.0) | 44 | 8.1 (4.1)  7.0  (4.0-7.0) |
| Negative symptoms (PANSS) | 46 | 11.2 (5.2)  10.0  (6.0-27.0) | 47 | 10.5 (4.6)  9.0  (6.0-25.0) | 44 | 10.8 (4.9)  8.5  (6.0-23.0) | 43 | 10.3 (4.6)  9.0  (6.0-25.0) | 39 | 10.6 (4.2)  10.0  (6.0-21.0) | 44 | 10.6 (5.0)  9.0  (6.0-29.0) |
| Disorganised symptoms (PANSS) | 46 | 8.1 (3.3)  7.5  (4.0-19.0) | 46 | 8.0  (2.6)  7.5  (4.0-16.0) | 45 | 7.4 (3.2)  7.0  (4.0-17.0) | 43 | 7.5 (3.0)  7.0  (4.0-20.0) | 38 | 6.8 (3.0)  6.0  (4.0-18.0) | 44 | 7.4 (2.7)  7.0  (4.0-17.0) |
